# A mouse model for evaluation of efficacy and concomitant toxicity of anti-human CXCR4 therapeutics

**DOI:** 10.1371/journal.pone.0194688

**Published:** 2018-03-19

**Authors:** Maria José Costa, Jyothirmayee Kudaravalli, Wen-Hui Liu, Jeffrey Stock, Sophanna Kong, Shu-Hui Liu

**Affiliations:** 1 Cancer Immunology Discovery, Oncology Research and Development, Worldwide Research and Development, Pfizer Inc., South San Francisco, California, United States of America; 2 Discovery Sciences, Medicinal Sciences, Worldwide Research and Development, Pfizer Inc., Groton, Connecticut, United States of America; University of Florida, UNITED STATES

## Abstract

The development of therapeutic monoclonal antibodies through mouse immunization often originates drug candidates that are not cross-reactive to the mouse ortholog. In such cases, and particularly in oncology, drug efficacy studies are performed on human tumor xenografts or with “surrogate” anti-mouse ortholog antibodies if targeting tumor host cells. Safety assessment of drug candidate(s) is performed at a later development stage in healthy non-human primates. While the latter remains necessary before a drug advances into human subjects, it precludes evaluation of safety in disease conditions and drug de-risking during early development. Therefore, mouse models that allow concomitant evaluation of drug efficacy and safety are highly desirable. The C-X-C motif chemokine receptor 4 (CXCR4) is an attractive target for tumor-targeted and immuno-oncology therapeutics, with multiple mouse immunization-derived antibodies undergoing clinical trials. Given the pleiotropic role of CXCR4 in cancer biology, we anticipate continuous interest in this target, particularly in the testing of therapeutic combinations for immuno-oncology. Here, we describe the generation and validation of the first mouse knock-in of the whole coding region of human CXCR4. Homozygous human CXCR4 knock-in (hereafter designated as HuCXCR4KI) mice were viable and outwardly healthy, reproduced normally and nursed their young. The expression pattern of human CXCR4 in this model was similar to that of CXCR4 expression in normal human tissues. The human CXCR4 knock-in gene was expressed as a biologically active protein, thereby allowing normal animal development and adequate”homing” of leukocytes to the bone marrow. To further validate our model, we used an *in vivo* functional assay of leukocyte mobilization from bone marrow to peripheral blood by blocking CXCR4 signaling. Both an anti-human CXCR4 -specific blocking antibody and the small molecule CXCR4 inhibitor AMD3100 induced increased leukocyte counts in peripheral blood, whereas an anti-mouse CXCR4 –specific blocking antibody had no effect. This new mouse model is useful to evaluate efficacy and safety of anti-human CXCR4 -specific drugs as single agents or in combination therapies, particularly in the oncology, immuno-oncology, wound healing and chronic inflammation therapeutic areas.

## Introduction

The chemokine receptor CXCR4 and its ligand, CXCL12, play a key role in governing progenitor cell migration during embryonic development and in the maturation of myeloid cells and B-lymphocytes [1]. Post birth, in normal physiological conditions, CXCL12/CXCR4 axis is also important for the homeostasis of hematopoietic stem cells and the retention of neutrophils and CD34^+^ hematopoietic progenitors in the bone marrow [2,3]. Outside of adult hematopoietic tissues, CXCR4 expression has been reported in human specimens of normal appearance of adrenal gland cortex, colon epithelium and renal tubular epithelium [4–6]. CXCR4 and CXCL12 are highly conserved between species and the homology between human and murine CXCR4 and CXCL12 is 91% and 99%, respectively, allowing CXCL12 to signal across the two species [7–9]. CXCR4 also plays key functions in pathological conditions. CXCR4 was identified as a CD4 co-receptor required for entry of T-tropic HIV-1 strains [10]. In cancer, the physiological roles of CXCL12/CXCR4 pathway can be co-opted to promote tumor growth and metastasis. Guided by CXCL12 concentration gradients, CXCR4^+^ hematological or solid tumor cells “home” to the bone marrow, wherein chemotherapy-induced cell death signals are counteracted by pro-survival growth factors. Besides bone marrow, CXCL12 is expressed by tissue-resident mesenchymal stromal cells in multiple organs. Consequently, solid tumor cells that have acquired CXCR4 expression can use this chemotaxis pathway for metastatic dissemination. In addition, bone marrow-derived CXCR4^+^ vascular cells can be recruited by CXCL12-expressing tumor/stromal cells and contribute to a microenvironment that supports tumor angiogenesis [7,11,12]. Thus, CXCR4 has been an attractive therapeutic target in several chronic indications, and various small molecule and peptide inhibitors were generated to target it for the management of HIV-1 infection and cancer [13–15]. However, these drugs often present unfavorable pharmacokinetic and toxicity profiles that limit their therapeutic application through chronic administration [16]. Nevertheless, single dose with small molecules blocking CXCR4 pathway proved useful in the mobilization of hematopoietic precursors. AMD3100 (Plerixafor/Mozobil), though originally designed for blocking HIV-1 docking on CXCR4, is now an approved therapy for CD34^+^ bone marrow-derived progenitor cell collection prior to hematopoietic stem cell transplantation [13,17–19]. To overcome the limitations of CXCR4-targeting with small molecules in cancer therapy, the new generation of therapeutics targeting CXCR4 is comprised of monoclonal antibody-based approaches. These antibodies are usually selected as human/primate-specific and the currently available therapeutic antibodies for human CXCR4 are not cross-reactive to the mouse ortholog [20–23].

*In vivo* pre-clinical models of drug efficacy in oncology research are usually restricted to mice, whereas safety studies are performed in healthy non-human primates. Often, it is not until candidate drugs are tested in the clinic that efficacy and safety can be evaluated in the same individual, frequently leading to poor safety profile and/or a lack of clinical activity within the tolerated dose, and consequently, high rates of drug attrition. To date, oncology pre-clinical *in vivo* studies for CXCR4-targeting antibodies consist of human CXCR4^+^ tumor cells implanted or infused in immunocompromised mice or the use of anti-mouse CXCR4 “surrogate” antibodies in the case of mouse syngeneic tumors and tumor-prone genetic models. Like other heptahelical receptors, CXCR4 is a challenging target for antibody generation. As such, surrogate antibodies are not always available and their activity and/or biophysical properties might differ from the human CXCR4-specific clinical candidate molecule(s). Given the important function of the CXCL12/CXCR4 signaling pathway for hematopoietic system homeostasis, and the complex roles it plays in the microenvironment of hematological and solid tumors, as well as in fibrosis, chronic inflammation and wound healing, we propose that a mouse model that allows simultaneous evaluation of efficacy and safety of anti-human CXCR4 therapeutics can prove very useful for de-risking early in the drug development process and for evaluation of potential treatment combinations. Herein, we describe the generation and validation of the first full length human CXCR4 knock-in mouse model.

## Materials and methods

### Knock-in mouse development

A mouse fosmid clone (WI1-2736E10) and a human BAC clone (RP11-1140A6) were used to construct the targeting vector using homologous recombination-based technique. We designed primers that contain the flanking genomic sequences for the iNeo cassette. The PCR product was used for bacterial homologous recombination (BHR) to insert the cassette into the hBAC. The hBAC was then sub-cloned and inserted into a mini-vector with flanking genomic sequences from the mouse, which was used for BHR into the mouse fosmid clone to replace mouse exon 1 through 2 (3,367bp), i.e., the human CXCR4 ATG-Stop codon replaced the mouse ATG-Stop codon. The recombinants were screened by colony PCR and confirmed by sequencing. We confirmed targeting vector junction regions by PCR and by sequencing after each BHR step. The final targeting vector was confirmed by sequencing and by restriction enzyme digestion. The total size of the final targeting vector including the hBAC sub-clone and the iNeo cassette is ~40kb. The targeting vector was linearized with NotI prior to electroporation into ES cells. We obtained two mouse lines with identical human CXCR4 expression in peripheral blood leukocytes of homozygous knock-in. We then proceeded to establish a colony with one of the lines. All mice characterized in this study were progeny of heterozygous (hereafter termed as HuCXCR4KI/WT) mating pairs and their age was 3–6 months. All studies with comparison between genotypes were performed with age- and gender- matched WT littermate controls. All animal studies were conducted in an AAALAC International accredited facility, under strict compliance with a CID-Pfizer Institutional Animal Care and Use Committee approved protocol (R.330.1–2016) and in accordance with the recommendations set in the Guide for the Care and Use of Laboratory Animals of the National Institutes of Health.

### Peripheral blood flow cytometry and leukocyte enumeration

Under isoflurane anesthesia (5% in 2 L/min. oxygen), peripheral blood was collected in K_2_EDTA tubes (BD Vacutainer) and mice were immediately euthanized by cervical dislocation. One hundred μL of whole blood was transferred to 5 mL FACS tubes (BD falcon) and 15 μL of CountBright absolute counting beads (Life tech) were spiked into each sample. Red blood cells were lysed with LCK lysing buffer at room temperature (Gibco) and white blood cells were washed in 0.5% bovine serum albumin in phosphate-buffered saline (0.5% BSA-PBS) and centrifuged at 300 x *g*, for 5 min. at 4°C. Cells were incubated with 1 μg anti-mouse CD16/CD32 (clone 93, eBioscience) in a total volume of 100 μL with 0.5% BSA-PBS for 5 min. on ice, and then a cocktail of antibodies (1 μg each in a total volume of 100 μL completed with 0.5% BSA-PBS) for analysis of leukocyte subsets. Antibodies used were: anti-CD19-PE-Cy7 (clone 1D3, eBioscience), anti-Thy1.2-AlexaFluor647 (clone 53–2.1, BD Biosciences), CD45-AlexaFluor700 (clone 30F-11, BD Biosciences), anti-CD49b-PerCP/Cy5.5 (clone DX5, Biolegend), anti-CD11b-pacific blue (clone M1/70, BD Biosciences), anti-CD115-APC-Cy7 (clone AFS98, Biolegend) and anti-Ly6G-FITC (clone 1A8, BD Biosciences). In experiments aimed at confirming expression of human CXCR4 knock-in, a proprietary anti-human/cynomolgus monkey CXCR4-specific antibody (Costa et al., *manuscript in preparation*) followed by goat anti-human Fc-specific antibody conjugated to AlexaFluor647 (Jackson ImmunoResearch) was used (1 μg each in a total volume of 100 μL with 0.5% BSA-PBS). After 20 min. incubation on ice with each antibody, cells were washed with 0.5% BSA-PBS, centrifuged at 300 x *g*, for 5 min. at 4°C, and re-suspended in 100 μL 0.5% BSA-PBS for data acquisition in a BD LSRFortessa (BD Biosciences). Data was analyzed using FlowJo (v10). For enumeration of blood cells, we used the formula: *Cell count/μL blood = [(Number of recorded single cells x Total number of spiked beads in 15 μL)/Number of recorded beads]/100 μL blood*

### Taqman analysis of human CXCR4 knock-in mRNA expression

Male HuCXCR4KI mice were euthanized by CO_2_ asphyxiation followed by cervical dislocation. Whole organs were harvested and approximately half sample snap-frozen in liquid nitrogen and stored at– 80°C for later processing. Organ samples were homogenized and total RNA isolated using RNeasy Plus Universal Midi Kit (Qiagen) and the FastPrep-24 tissue homogenizer with lysing matrix A (MP Biomedicals). Integrity of RNA samples was checked using Agilent 2200 TapeStation System. Total RNA (350 ng) was random primed with hexamers and retro-transcribed using SuperScript IV cDNA synthesis kit (Invitrogen). Taqman was performed with 100 ng cDNA in a final reaction volume of 20 μL (Life Tech). Expression of mouse ribosomal protein L19 (*Rpl19*) was used as a normalization control. Complementary DNA synthesis reactions with liver-derived total RNA samples in which reverse transcriptase was omitted were used as genomic DNA amplification control.

### Immunohistochemistry

For immunohistochemical detection of human CXCR4 in both human specimens and HuCXCR4KI organ samples (from same mice as used in mRNA expression studies), we used a previously validated antibody (clone UMB2, Abcam) [4]. Formalin-fixed, paraffin-embedded (FFPE) HuCXCR4KI tissues were sectioned to 4μm. FFPE sections of human normal tissue microarrays (MNO381, MNO961 and MNO1021) were purchased from US BioMax. Antigen retrieval was performed in DIVA HIER pH 6.0 buffer (Biocare Medical) for human TMA samples and rodent HIER pH 6.0 buffer (Biocare Medical) for mouse samples, both at 110°C, 5 PSI for 15 min. After blocking of endogenous peroxidases with 3% hydrogen peroxide and of non-specific binding with protein block (Agilent), sections were incubated with anti-CXCR4 antibody (5μg/ml) at 4°C, overnight. HRP-labelled polymer anti-rabbit antibody (Envision system+, DAKO) was applied on sections for 30 min. at room temperature and visualized with 3,3’-diaminobenzidine (DAKO). Sections were counterstained with hematoxylin (Vector Labs), dehydrated and mounted with Permount.

### ELISA of serum CXCL12

Under isoflurane anesthesia (5% in 2 L/min. oxygen), peripheral blood was collected in serum separator tubes (BD Vacutainer) and mice were immediately euthanized by cervical dislocation. Serum samples were analyzed for CXCL12 levels using mouse CXCL12/SDF-1 alpha Quantikine ELISA kit (R&D systems).

### Leukocyte mobilization assay

AMD3100 Octahydrochloride (Sigma-Aldrich) was solubilized in phosphate-buffered saline (PBS) and administered to HuCXCR4KI female mice through a single intra-peritoneal (i.p.) injection (5 mg/kg) [24]. An equivalent dosing volume of PBS (100 μL) was injected i.p. in the animals of the vehicle control treatment group. All treatment antibodies were prepared in PBS and dosed through the tail vein in a single 100 μL injection. HuCXCR4KI female mice received anti-human CXCR4 -specific human IgG2Δa or isotype non-target antibody control (both proprietary to Pfizer, Costa et al., *manuscript in preparation*) or anti-mouse CXCR4 –specific antibody (clone L276F12, Biolegend), at the doses indicated in the figure legends. Wild type Balb/c female mice were dosed with 10 mg/kg of anti-mouse CXCR4 –specific antibody (clone L276F12, Biolegend) or non-target control antibody. Under isoflurane anesthesia (5% in 2 L/min. oxygen), peripheral blood was collected in K_2_EDTA tubes (BD Vacutainer), 1 hour post-dose, and mice were immediately euthanized by cervical dislocation. One hundred μL of whole blood was processed for flow cytometry using anti-CD45-BV786 (clone 30F-11, BD Biosciences) to label leukocytes, and their number was estimated using absolute counting beads, as described above.

## Results

HuCXCR4KI and wild type (WT) littermate mice were generated from inter-crosses of HuCXCR4KI/WT mating pairs in the C57Bl6 background. Both HuCXCR4KI/WT and HuCXCR4KI mice were fertile, and females nursed their litters, indicating normal post-birth development. HuCXCR4KI were outwardly healthy and undistinguishable from age- and gender- matched WT mice. We did notice that both male and female HuCXCR4KI mice displayed a trend for lower body weight than WT counterparts, though the difference did not reach statistical significance.

**Fig 1:**
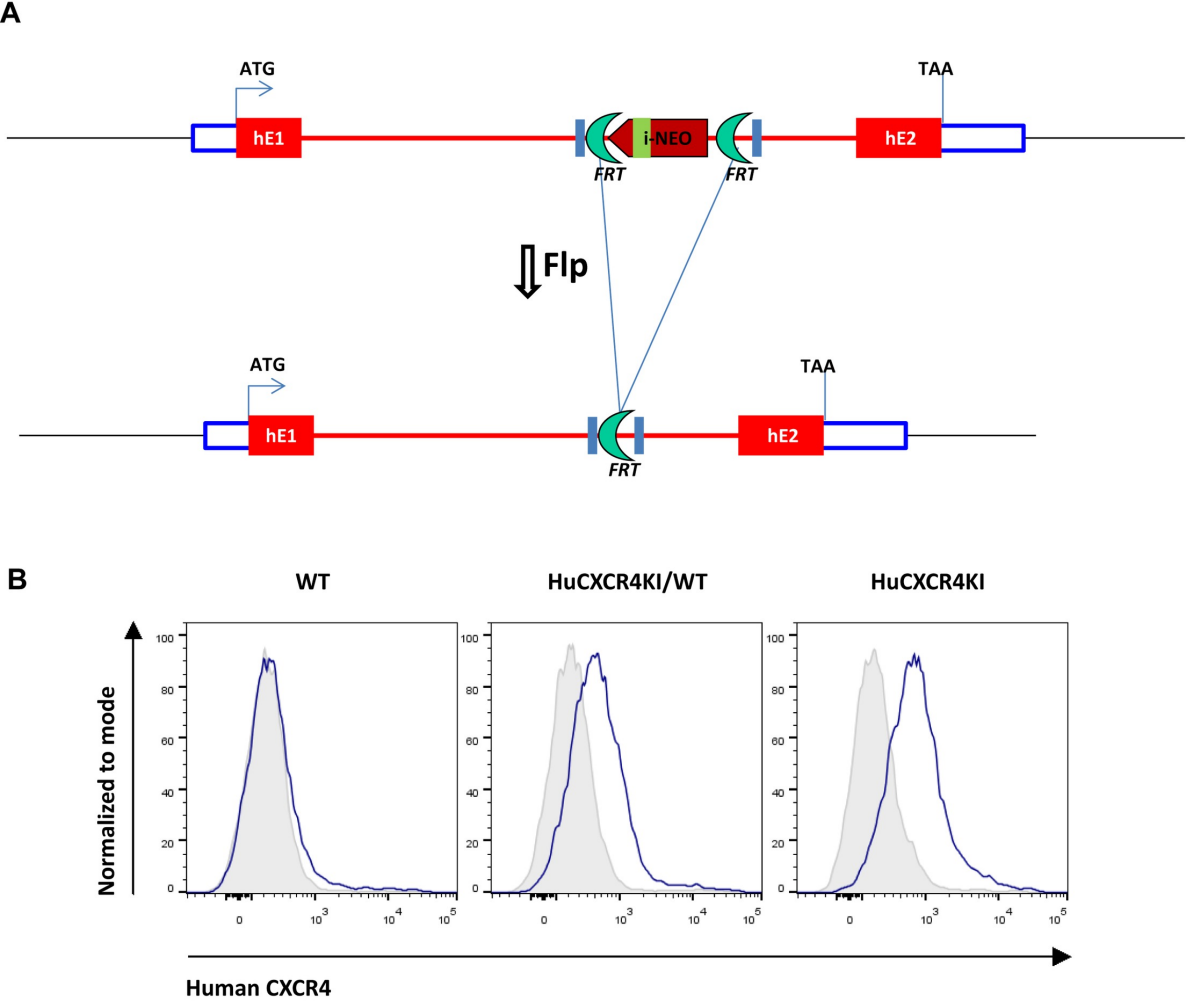
Human CXCR4 knock-in design and confirmation of human CXCR4 expression in peripheral blood cells of heterozygous (HuCXCR4KI/WT) and homozygous (HuCXCR4KI) mice. **A**, Schematic of genetic targeting strategy for knock-in of full length human CXCR4 coding region. **B**, Flow cytometric analysis of peripheral blood leukocytes from littermate mice with antibodies specific for human CXCR4.

In HuCXCR4KI mice, the expression of full-length human CXCR4 is under control of the mouse promoter/enhancer (**Fig 1A**). As such, human CXCR4 expression pattern will be the same as that of mouse CXCR4. Given that the ligand for CXCR4—CXCL12 –is 99% homologous between human and mouse and functional across species, it is highly likely that mouse CXCL12 can signal through human CXCR4 in this animal model [7,9]. Given that signal transduction by CXCR4 involves a continuous intramolecular signaling chain through the receptor transmembrane helices, our knock-in model has an advantage in that the human CXCR4 amino acid sequence is kept intact, thereby ensuring proper signal transduction [25]. This could potentially not be the case with a chimeric CXCR4 molecule comprising human extracellular domains and mouse transmembrane helices and cytoplasmic regions. Several global knock-out mice for CXCR4 have been described and are embryonic lethal due to developmental vascular, cerebellar and hematopoietic defects [26–28]. Therefore, the successful generation of outwardly normal HuCXCR4KI mice suggests competent mouse CXCL12 -to- human CXCR4 signaling.

In adult mice and humans, CXCR4 expression is maintained in leukocytes. To confirm the replacement of mouse CXCR4 gene by human CXCR4 gene in HuCXCR4KI mice, we evaluated surface CXCR4 expression in peripheral blood leukocytes of WT, HuCXCR4KI/WT and HuCXCR4KI mice, using antibodies that are specific for human CXCR4. We found that these antibodies bind peripheral blood leukocytes from HuCXCR4KI, but not leukocytes from WT mice. Intermediate staining signal for human CXCR4 was observed in leukocytes of HuCXCR4KI/WT mice (**Fig 1B**).

**Fig 2:**
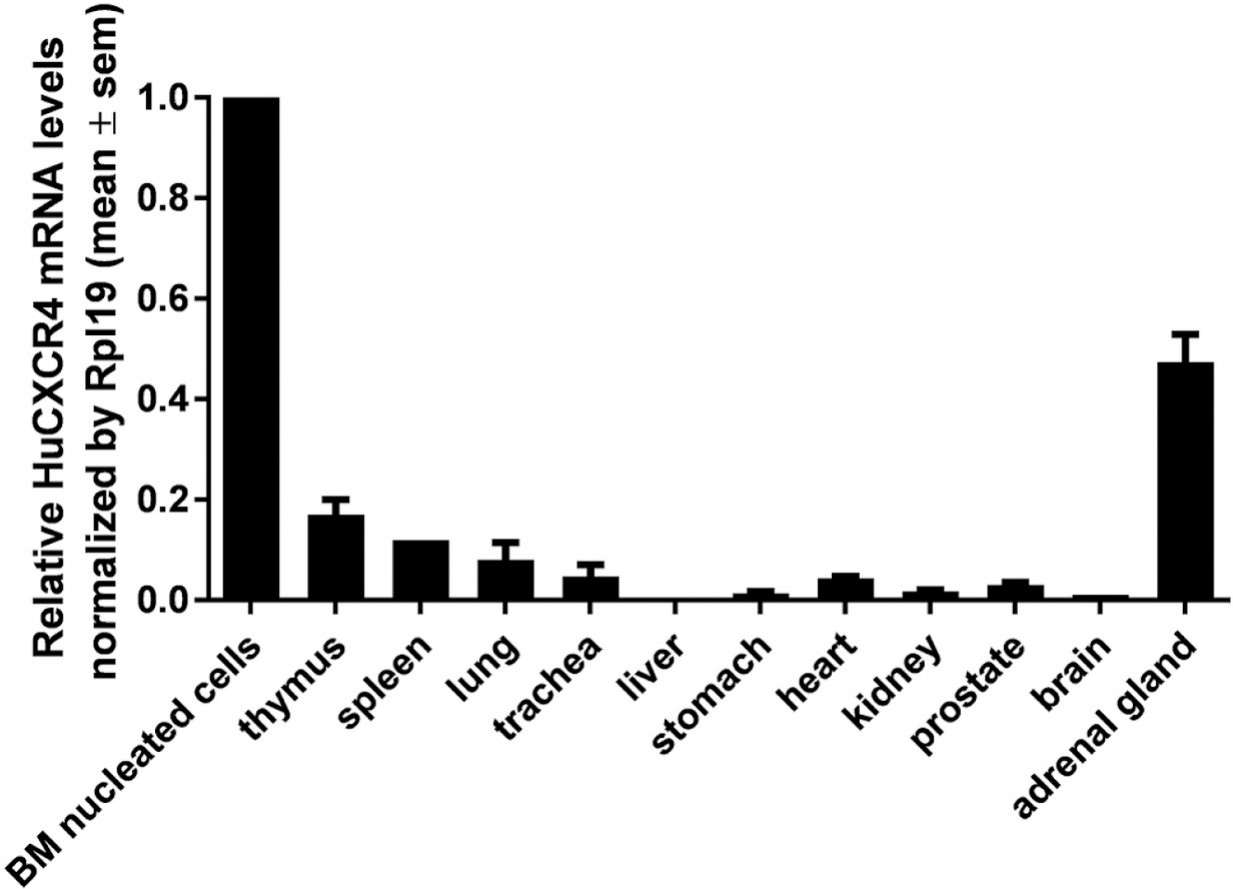
Expression of human CXCR4 mRNA is detectable in various organs of HuCXCR4KI mice. Relative expression levels of human CXCR4 mRNA in whole organs of HuCXCR4KI male mice, as detected by RT-qPCR (Taqman). Equal amounts of total RNA were retro-transcribed and the levels of mouse Rpl19 transcript were used as a normalization control.

To characterize CXCR4 expression in multiple tissues of HuCXCR4KI mice, we performed reverse transcription and Taqman analysis in several organ samples with probe and primers for human CXCR4. Among analyzed tissues, we found the highest levels of RNA transcript expression in femur bone marrow nucleated cells and in the adrenal gland. Lower expression was observed in thymus, spleen and lung. Very low expression levels were detected in the heart, kidney, stomach, and prostate, whereas no expression was detected in the brain or liver (**Fig 2**).

**Fig 3:**
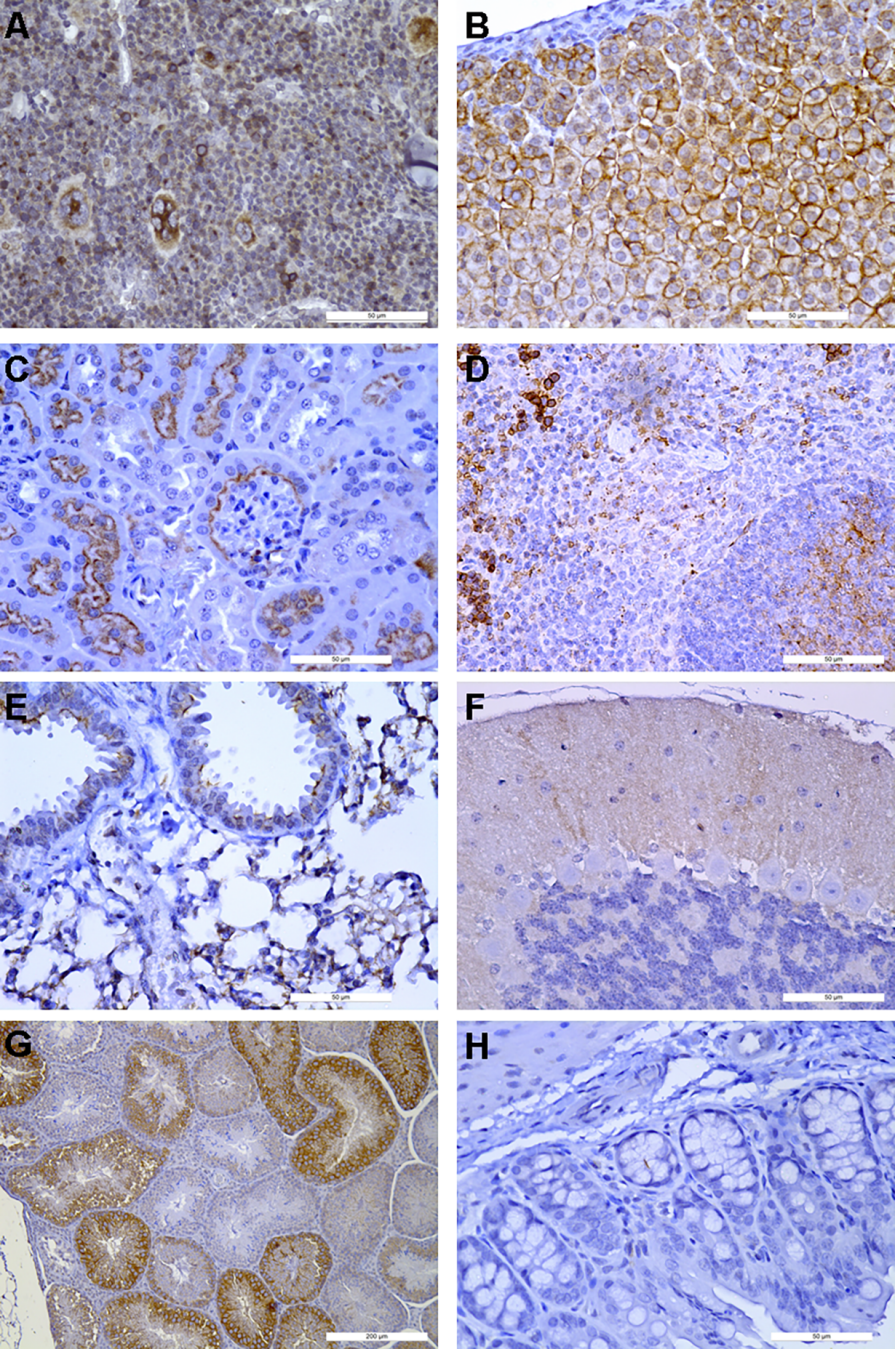
Immunohistochemical detection of human CXCR4 in organs of HuCXCR4KI mice. **A**, bone marrow. **B**, adrenal gland. **C**, kidney. **D**, Spleen. **E**, lung. **F**, cerebellum. **G**, testis. **H**, colon.

We then aimed to confirm protein expression in parallel tissue samples of the same mice as analyzed in Fig 2 by immunohistochemistry with a previously validated anti-CXCR4 antibody [4]. We observed CXCR4 protein expression in bone marrow nucleated cells (namely, in hematopoietic precursors, including megakaryocytes), in the adrenal gland, and in tubular epithelial cells of the kidney (**Fig 3A–3C**). CXCR4 expression was also found in some splenocytes, lung alveoli and in the cerebellum (**Fig 3D–3F**). CXCR4 protein was also detected in germ cells in the testis, but this tissue was not analyzed for CXCR4 mRNA expression (**Fig 3G**). CXCR4 protein expression was undetectable in the colon, liver, pancreas, thymus or heart. (**Fig 3H** and **Table 1**). In the stomach and small intestine, CXCR4 was detected in infiltrating cells (**Table 1**).

**Table 1:** Survey of human CXCR4 expression in TMA of human specimens of normal appearance and in corresponding tissues of homozygous HuCXCR4KI mice.

Organ	Fraction of human specimens with detectable CXCR4 expression	Fraction of HuCXCR4 specimens with detectable CXCR4 expression
**Adrenal gland**	6/6	3/3
**Bladder**	1/5	n/a
**Bone marrow**	1/3	3/3
**Cerebellum**	3/16	3/3
**Esophagus**	1/8	3/3
**Colon**	2/12	0/3
**Kidney**	3/12	3/3
**Tonsil**	8/8	n/a
**Pituitary gland**	4/4	n/a
**Stomach**	0/7	3/3
**Small intestine**	0/8	0/3
**Heart**	0/8	0/3
**Liver**	0/8	0/3
**Lung**	0/8	3/3
**Ovary**	0/8	n/a
**Pancreas**	0/8	0/3
**Parathyroid**	0/2	n/a
**peripheral nerve**	0/2	n/a
**Prostate**	0/8	0/3
**Skin**	0/7	3/3
**Spleen**	0/5	3/3
**Skeletal muscle**	0/6	0/3
**Testis**	0/8	3/3
**Thyroid**	0/8	n/a
**Thymus**	0/5	0/3
**Ureter**	0/4	n/a
**Cervix**	0/2	n/a
**Uterus**	1/10	n/a
**Placenta**	0/6	n/a
**Breast**	0/7	n/a
**Fallopian tube**	0/6	n/a

n/a = not analyzed.

**Fig 4:**
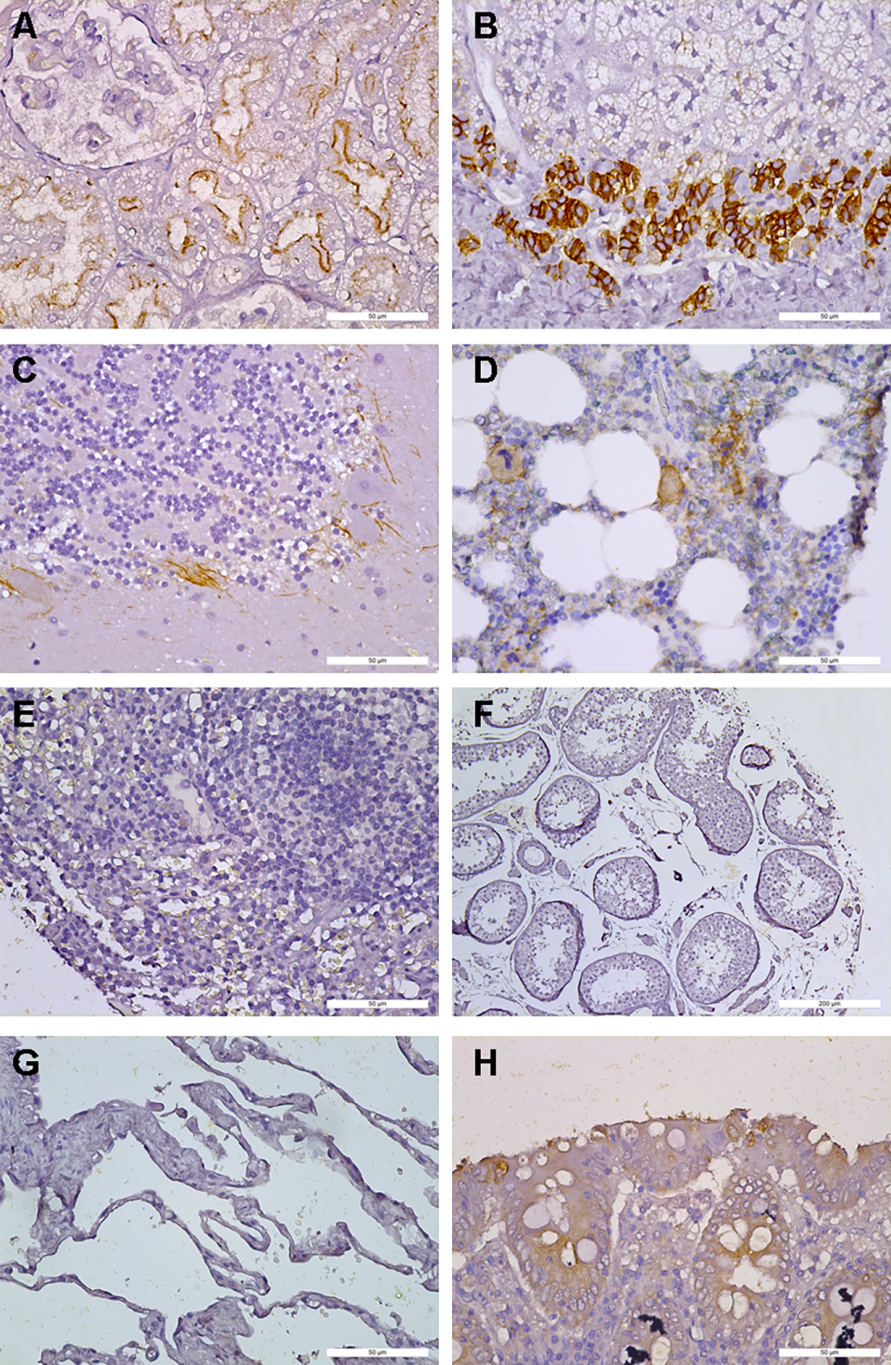
Immunohistochemical detection of CXCR4 expression in human tissue specimens of normal appearance. **A**, kidney. **B**, adrenal gland. **C**, cerebellum. **D**, bone marrow, brown staining: CXCR4, green staining: CD45. **E**, Spleen. **F**, testis. **G**, lung. **H**, colon.

To compare human CXCR4 expression pattern between HuCXCR4KI and human specimens and to evaluate the usefulness of HuCXCR4KI as a tool for pharmacological studies targeting human CXCR4, we surveyed CXCR4 expression by immunohistochemistry in three tissue microarrays (TMA) of human specimens of normal appearance. Similar to what we observed in HuCXCR4KI mice, we found CXCR4 protein expression in the kidney tubular epithelium, adrenal gland, cerebellum and nucleated hematopoietic cells of bone marrow (**Fig 4A–4D**). We also found that a subset of cells in human pituitary gland expressed CXCR4, but this organ was not analyzed in the HuCXCR4KI mice (**Table 1**). Contrary to what was observed in HuCXCR4KI mice, CXCR4 expression was undetectable in the human spleen, testis and lung alveoli (**Fig 4E–4G**). With the exception of the colon, in which weak and cytoplasmic diffuse signal was detected in epithelial cells of 2/12 human specimens (**Fig 4H**), but not in colonic epithelium of HuCXCR4KI mice (**Fig 3H**), all other human tissues with detectable CXCR4 expression were matched with similar expression pattern in corresponding tissues of HuCXCR4KI (**Fig 3** and **Table 1**). CXCR4 was also expressed in the human tonsil (**Table 1**), but this tissue is absent in mice [29]. As expected, most analyzed human and HuCXCR4KI tissues did not show immunoreactivity for CXCR4 (**Table 1**).

**Fig 5:**
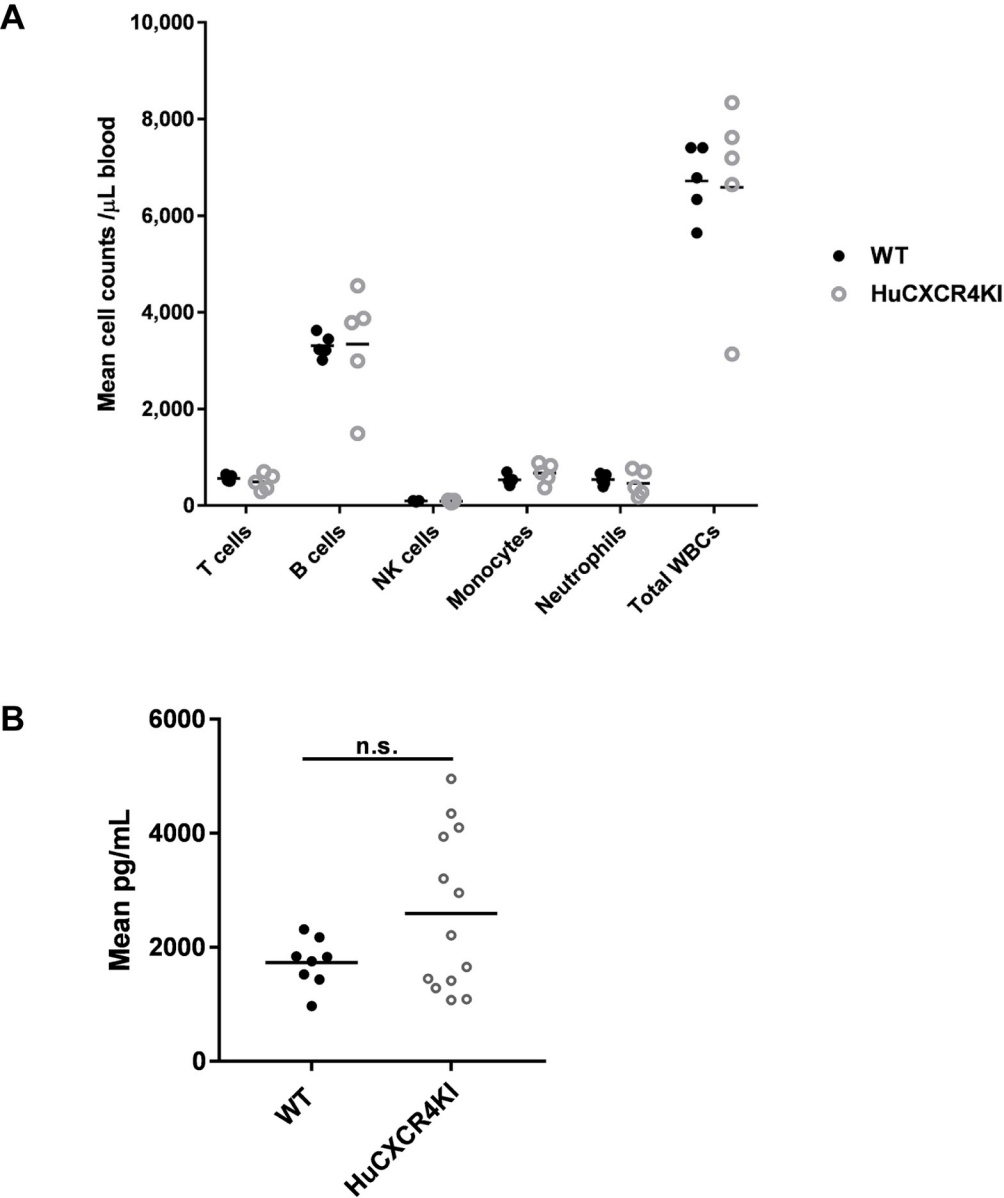
Normal blood cell counts in peripheral blood and normal CXCL12 levels in serum indicate proper CXCL12/CXCR4 signaling in HuCXCR4KI mice. Each symbol represents the result from an individual mouse. **A**, Flow cytometric analysis of total leukocyte (WBCs) and leukocyte subset counts in peripheral blood of HuCXCR4KI and WT control littermate mice. Cell counts were estimated with absolute counting beads, as described in the methods section. WBCs were defined as CD45^+^ blood cells. WBC subsets were defined based on cell surface marker expression, as follows: monocytes (CD45^+^CD11b^+^CD115^+^Ly6G^-^), neutrophils (CD45^+^CD11b^+^CD115^-^Ly6G^+^), T cells (CD45^+^CD11b^-^Thy1.2^+^), B cells (CD45^+^CD11b^-^CD19^+^), NK cells (CD45^+^CD11b^-^CD49b^+^). **B**, Detection by ELISA of CXCL12 levels in the serum of HuCXCR4KI and WT control littermate mice; n.s. = non-significant (unpaired Student’s *t*-test).

Given the important role played by CXCR4 in the homeostasis of hematopoietic precursors, and in the retention of hematopoietic precursors and neutrophils in the bone marrow [1,2], we sought to determine whether counts of leukocyte subsets were normal in peripheral blood of HuCXCR4KI mice. Both total leukocyte and leukocyte subset counts were similar between HuCXCR4KI and WT mice, suggesting normal hematopoiesis in HuCXCR4KI mice (**Fig 5A**). In addition, we found that the levels of CXCL12 in the serum of HuCXCR4KI mice, though variable among animals, were comparable to those of WT mice (**Fig 5B**).

**Fig 6:**
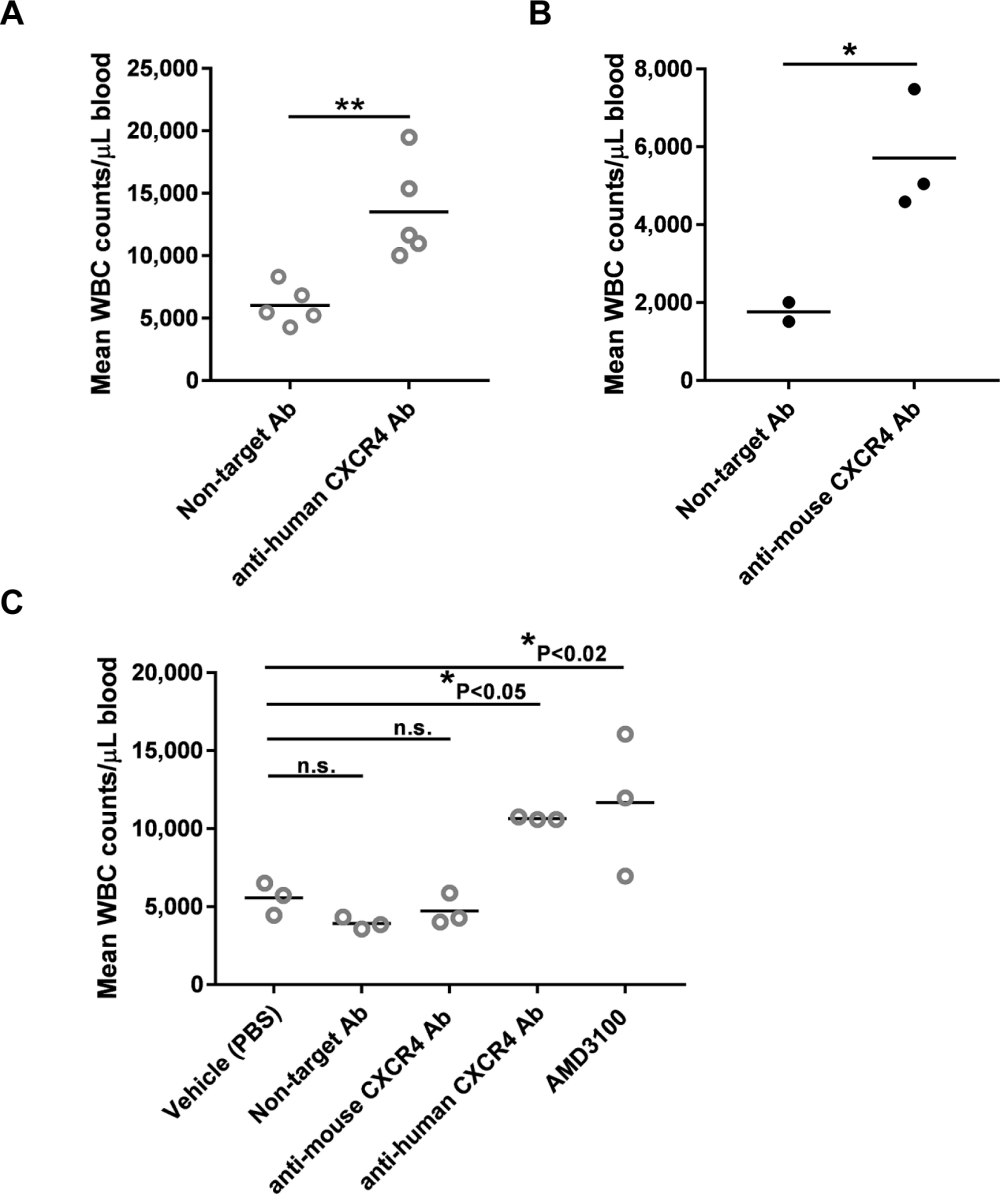
Increased leukocyte count in peripheral blood of HuCXCR4KI mice upon dosing with a blocking antibody specific for human CXCR4 indicates functional signaling between mouse CXCL12 and human CXCR4 and validates the HuCXCR4KI mouse model for testing of anti-human CXCR4 -specific therapeutics. *In vivo* functional assays of leukocyte (WBC) mobilization from bone marrow into peripheral blood mediated by CXCR4 signaling blockade. Mice were administered with the indicated treatments and peripheral blood samples collected after 1 hour for leukocyte enumeration, as described in the methods section. Each symbol represents the result from an individual mouse. **A**, HuCXCR4KI mice were dosed with 3 mg/kg of either anti-human CXCR4 -specific human IgG2Δa or isotype non-target control antibody, ** P = 0.004 (unpaired Student’s *t*-test). **B**, As a reference for the magnitude of leukocyte mobilization in HuCXCR4KI mice, we show data from an independent experiment, in which Balb/c wild type mice were dosed with either 10 mg/kg anti-mouse CXCR4 -specific or non-target control antibody, * P < 0.05, (unpaired Student’s *t*-test). **C**, HuCXCR4KI mice were dosed with 1 mg/kg of anti-human CXCR4 -specific human IgG2Δa, or 1 mg/kg isotype non-target antibody control, or 1 mg/kg anti-mouse CXCR4 –specific antibody or 5 mg/kg AMD3100, or PBS. Data was analyzed using ANOVA with Dunnett’s multiple comparisons test, relative to PBS treatment control group, n.s. = non-significant.

As mentioned above, CXCL12/CXCR4 signaling mediates hematopoietic cell retention in the bone marrow. Our results showing normal neutrophil counts in the peripheral blood of HuCXCR4KI mice suggest proper retention in bone marrow, mediated by functional mouse CXCL12 -to- human CXCR4 signaling. Blocking CXCR4 with pharmacological agents disrupts CXCL12 signaling and leads to leukocyte release and increased white blood cell (WBC) count in peripheral blood (leukocytosis). Therefore, to further ascertain whether CXCL12/CXCR4 signaling is functional in HuCXCR4KI mice, these were treated with an anti-human/cynomolgus CXCR4 -specific blocking antibody that lacks active Fc-mediated effector function (human IgG2Δa) (Costa et al., *manuscript in preparation*). One hour post-dose, we found a significant 2.2-fold increase in WBC count which reached clinical threshold for leukocytosis (>10,000 WBC/μL). In one control, WBC counts in HuCXCR4KI mice treated with isotype non-target control antibody were similar to WBC counts in untreated mice of both HuCXCR4KI and WT genotypes (**Fig 6A**, see also **Fig 5A**). The magnitude of leukocyte mobilization caused by the anti-human CXCR4 -specific blocking antibody in HuCXCR4KI mice was comparable to that observed in an independent experiment in which wild type Balb/c mice were treated with an antibody specific for mouse CXCR4, L276F12 (**Fig 6B**). This result indicates that L276F12 blocks mouse CXCR4 signaling *in vivo*. AMD3100 (Plerixafor/Mozobil) is clinically used for hematopoietic cell mobilization from bone marrow to peripheral blood and inhibits both human and mouse CXCR4 signaling [24,30]. In humans, a single AMD3100 injection produces a transient 1.5–3 fold increase in WBC counts in peripheral blood, peaking at 6 hours post dose [30]. In Balb/c mice, a 2-fold rise in peripheral blood WBC counts upon 5 mg/kg AMD3100 i.p. injection peaks at 1 hour post dose [24]. To further validate HuCXCR4KI mice, we treated them with 5 mg/kg AMD3100 or with 1 mg/kg of blocking antibodies specific for either human CXCR4 or mouse CXCR4. Both AMD3100 and anti-human CXCR4 –specific antibody treatments elicited a 2-fold increase in WBC counts in peripheral blood 1 hour after dose (**Fig 6C**). In contrast to its leukocytosis effect in Balb/c mice (**Fig 6B**), the anti-mouse CXCR4 –specific blocking antibody did not induce any detectable WBC mobilization in HuCXCR4KI mice (**Fig 6C**). These results further demonstrate replacement of mouse CXCR4 by human CXCR4 in HuCXCR4KI mice.

## Discussion

In this report we describe the development, characterization and validation of the first animal model for the evaluation of efficacy and concomitant toxicity/safety of therapeutics targeting human CXCR4. To our knowledge, this is the first human CXCR4 knock-in mouse model available. In addition, we also present the most comprehensive analysis of CXCR4 protein expression in normal human and mouse tissues reported to date. Because in HuCXCR4KI, only the human CXCR4 coding region was inserted in place of its mouse ortholog, the human CXCR4 expression is under control of the mouse promoter/enhancer. Therefore, the knocked-in human CXCR4 gene will display the same expression pattern as the mouse CXCR4. Given that human and mouse CXCL12 share extremely high homology, the normal CXCL12-CXCR4 interaction should remain intact [9,10]. One advantage of this design is that any potential difference in expression pattern between mouse and human would not compromise normal animal development. In addition, it has now been demonstrated that CXCR4 activation involves intramolecular signaling relay throughout the entire molecule [25]. Therefore, keeping the human CXCR4 amino acid sequence intact instead of replacing only the extracellular domains with human sequences might contribute to proper signal transduction through this critical pathway. CXCR4 and CXCL12 are 91% and 99% homologous between human and mouse, respectively, and mouse CXCL12 is known to function across the two species [7–9]. All three intracellular loops are 100% homologous between human and mouse CXCR4. The CXCR4 intracellular C-terminus region is 94% homologous between human and mouse, and all serine residues that are predicted phosphorylation sites are conserved. Accordingly, our data shows that CXCL12 levels in the serum of HuCXCR4KI are within normal (WT) range, suggesting no pathway deficiency. In addition, the counts of leukocyte subsets in peripheral blood are also comparable to those of WT mice, suggesting both normal hematopoiesis and adequate leukocyte retention in the bone marrow. Moreover, the normal development of HuCXCR4KI mice suggests that functions of CXCL12/CXCR4 signaling outside of the hematopoietic compartment were also not impaired by the human knock-in.

The magnitude of leukocyte mobilization in response to CXCR4 blockade by an anti-human CXCR4 -specific antibody further indicates that human CXCR4 expression in hematopoietic cells mediates mouse CXCL12-driven retention of cells in bone marrow. The result of this experiment also validates the use of this model for the purpose of testing anti-human CXCR4 therapeutics, as leukocyte mobilization from bone marrow into peripheral blood is a hallmark of CXCR4 blocking agents [15].

Differences in CXCR4 expression between tissues of HuCXCR4KI mice and human tissues have also been addressed in this study, with the exception of female reproductive system and pituitary, which were not analyzed in HuCXCR4KI mice. CXCR4 expression has been reported in the rat pituitary and in human pituitary tumor cells, but within the human pituitary specimens of normal appearance analyzed in this work, only a minority of cells displayed detectable CXCR4 antibody reactivity [31,32]. On the other hand, none of the human female reproductive system tissues analyzed in our study displayed detectable CXCR4 expression. Among the tissues analyzed in both species, with exception of the colon, spleen and germ cells in testis, we present evidence that the human CXCR4 knock-in is expressed in the same tissue types as normal endogenous human CXCR4. It is important to establish this comparison, given that one main purpose of this model is the evaluation of normal tissue toxicity of candidate CXCR4 targeting agents at early stages of drug development. Our results of CXCR4 expression in the tubular epithelium of human kidney are in agreement with data from a previous report [33]. Our finding that CXCR4 is expressed in human adrenal gland is also corroborated in the literature [4]. CXCR4 expression in mouse spermatogonial stem cells has been reported elsewhere, but conflicting data on CXCR4 expression in human testes has been published [34–36]. Our data rather suggests that CXCR4 plays a role in development of mouse, but not human, sperm cells. CXCR4 expression in human colon epithelium of normal appearance has been reported by others, though in our study it was only detected in 16% of human specimens [5]. While we were able to standardize fixation conditions for the HuCXCR4KI FFPE tissue samples, we cannot rule out the possibility that lack of CXCR4 antibody reactivity in some human specimens could be due to unfavorable tissue fixation conditions. We tried to mitigate this factor by analyzing several TMA specimens for each tissue in our immunohistochemistry studies.

In oncology research, the safety profile of a drug candidate obtained in a healthy animal model might be modified by the presence of a tumor. This is particularly pertinent for targets expressed in hematopoietic cells, because the tumor not only influences its local microenvironment, but also elicits a systemic response that affects the homeostasis of primary and secondary lymphoid tissues. In this context, we anticipate that HuCXCR4KI can be very useful, because they allow co-evaluation of efficacy, exposure, and toxicity of anti-human CXCR4 -specific drug candidates. Our model is readily suitable for evaluating the activity of anti- human CXCR4 agents in host-derived tumor microenvironment cells and/or in immuno-oncology applications using syngeneic mouse tumor-derived cell lines. HuCXCR4KI are also useful to evaluate the potential efficacy/toxicity of combination treatments involving the anti-CXCR4 therapeutics previously tested in the clinic as monotherapies. If desired, HuCXCR4KI can be crossed to tumor-prone genetically engineered mouse models in the same C57Bl6 background to potentially generate tumors expressing human CXCR4 in both tumor and host cells. For growth of non-syngeneic tumors, and of human origin tumors in particular, one can also cross HuCXCR4KI mice with recombination activating genes (Rag1 or Rag2) knockout mice (both commercially available in the same C57Bl6 background) to eliminate adaptive anti-tumor immunity and allow tumor establishment.

## Conclusions

Here, we present the HuCXCR4KI mouse model as a novel, validated and useful tool for the simultaneous evaluation of efficacy and toxicity/safety of human CXCR4-specific treatments as monotherapies or in combination. The model can be readily used to study the effects of targeting CXCR4 in various therapeutic areas, such as chronic inflammation, oncology, immuno-oncology, fibrosis and wound healing. HuCXCR4KI mice are useful in the estimation of therapeutic indices and thus de-risking of drug candidates early in the drug discovery process.
